# Laser-sound reproduction by pulse amplitude modulation audio streams

**DOI:** 10.1038/s41598-024-62382-8

**Published:** 2024-05-27

**Authors:** Konstantinos Kaleris, Panagiotis Hatziantoniou, Bjoern Stelzner, Dimosthenis Trimis

**Affiliations:** 1 Wire Communications Laboratory, Audio and Acoustic Technology Group, Department of Electrical and Computer Engineering, University of Patras, 26500 Rio, Patras, Greece; 2 Institute of Plasma Physics and Lasers, Hellenic Mediterranean University, 74100 Rethymno, Greece; 3 Engler-Bunte-Institute, Karlsruhe Institute of Technology, Karlsruhe, Germany

**Keywords:** Photoacoustics, Electrical and electronic engineering, Laser-produced plasmas

## Abstract

Recently, the possibility to reproduce complex continuous acoustic signals via pulsed laser-plasma sound sources was demonstrated. This was achieved by optoacoustic transduction of dense laser pulse trains, modulated via single- or multi-bit Sigma–Delta, in the air or on solid targets. In this work, we extend the laser-sound concept to amplitude modulation techniques. Particularly, we demonstrate the possibility of transcoding audio streams directly into acoustic pulse streams by analog pulsed amplitude modulation. For this purpose, an electro-optic modulator is used to achieve pulse-to-pulse amplitude modulation of the laser radiation, similarly to the multi-level Sigma–Delta method. The modulator is directly driven by the analog input stream through an audio interface. The performance of the system is evaluated at a proof-of-principle level for the reproduction of test audio signals such as single tones, double tones and sine sweeps, within a limited frequency range of the audible spectrum. The results are supported by computational simulations of the reproduced acoustic signals using a linear convolution model that takes as input the audio signal and the laser-generated acoustic pulse profile. The study shows that amplitude modulation allows for significant relaxation of the laser repetition rate requirements compared to the Sigma–Delta-based implementation, albeit at the potential cost of increased distortion of the reproduced sound signal. The nature of the distortions is analyzed and a preliminary experimental and computational investigation for their suppression is presented.

## Introduction

Sound generation by laser induced breakdown (LIB) in ambient air or by laser ablation (LA) on solid targets by short or ultrashort laser pulses has been known since the early 60s^1,2^, shortly after the invention of the laser. Since then, laser-plasma sound generation has attracted significant scientific interest and has been extensively studied both experimentally and theoretically^3–6^. Laser-plasma sound sources (LPSSs) exhibit high practical interest for technological and scientific applications from the macro-scale to the micro-scale and from the very low frequencies up to the ultrasounds^6^. Point-like sources generated by tightly focused nanosecond laser pulses exhibit perfect omnidirectional emission across the entire frequency range, while line-like sources generated by loosely focused femtosecond pulses exhibit a cylindrical acoustic emission^7,8^. In the time domain, LPSSs have a rapid N-pulse pressure profile and a broadband and highly repeatable frequency spectrum^6^. Finally, LPSSs are effectively massless and spatially unbound, so that they can be reproduced over long distances without the need for in-situ power supply, receiver or demodulation devices.

Over the years, laser technology has evolved to develop laser systems capable of emitting high-energy short and ultrashort pulses at a broad range of wavelengths, durations and energies. Nanosecond, picosecond and femtosecond lasers capable of inducing breakdown in ambient air or other gases have become compact and affordable, enabling the adoption of LPSSs in scientific and industrial applications, such as laser-induced breakdown spectroscopy (LIBS)^9^, non-destructive materials testing and diagnostics^10,11^, underwater or air–water communication signal transmission^12^ and military applications^7^, while it has also been proposed for acoustic measurements^13,14^.

In previous works, we demonstrated the possibility to generate arbitrary complex and continuous acoustic signals through laser-plasma optoacoustic transduction by utilizing digital pulsed modulations, such as Sigma–Delta (ΣΔ) modulation^15,16^. Based on this concept, Lengert et al. have produced tones through laser-induced breakdown in gels using a similar pulse density modulation (PDM) scheme^17^. In this work, we extend laser-sound reproduction to pulse amplitude modulation (PAM). Analog or digital PAM schemes are adopted in telecommunications and audio technology with the most prominent applications being the Ethernet and digital television communications protocol^18^. PAM has also been adopted in the audio technology for the development of class-D amplifiers and for the conversion of voice signals into PCM signals^18^, among others. Here we demonstrate that PAM constitutes an attractive alternative to the digital ΣΔ-based implementation, mainly because it significantly relaxes the requirements for relatively high laser repetition rates. While ΣΔ modulation requires excessively dense pulse trains to encode information in the variable pulse-to-pulse density, PAM encodes information in the varying amplitude of the pulses at a repetition rate of the order of the Nyquist frequency of the input signal. As was shown in^15,16^, laser repetition rate is a crucial factor for system cost, energy efficiency and bandwidth. However, it is shown that the benefit of lower repetition rate comes at a cost of increased susceptibility to harmonic distortion and noise. These aspects are here analyzed and it is shown that the non-linearities mainly originate in the nonlinear relation between laser pulse energy and the induced acoustic pulse energy, while the noise mainly originates in the compromised repeatability of acoustic pulse reproduction due to the laser energy fluctuation and the use of a metal target. It is concluded that, although PAM-driven laser-sound could be difficult to use for high fidelity audio reproduction under real conditions, it could be highly beneficial for, but not limited to, applications where fidelity can be compromised for the sake of reduced laser system specifications. Such applications include audio, i.e., speech transmission over long distances, for example to remote or inaccessible locations, reproduction and broadcast of ultra-strong alarm signals and characterization/testing of acoustic structures, among others.

### Laser-plasma sound sources

**Figure 1:**
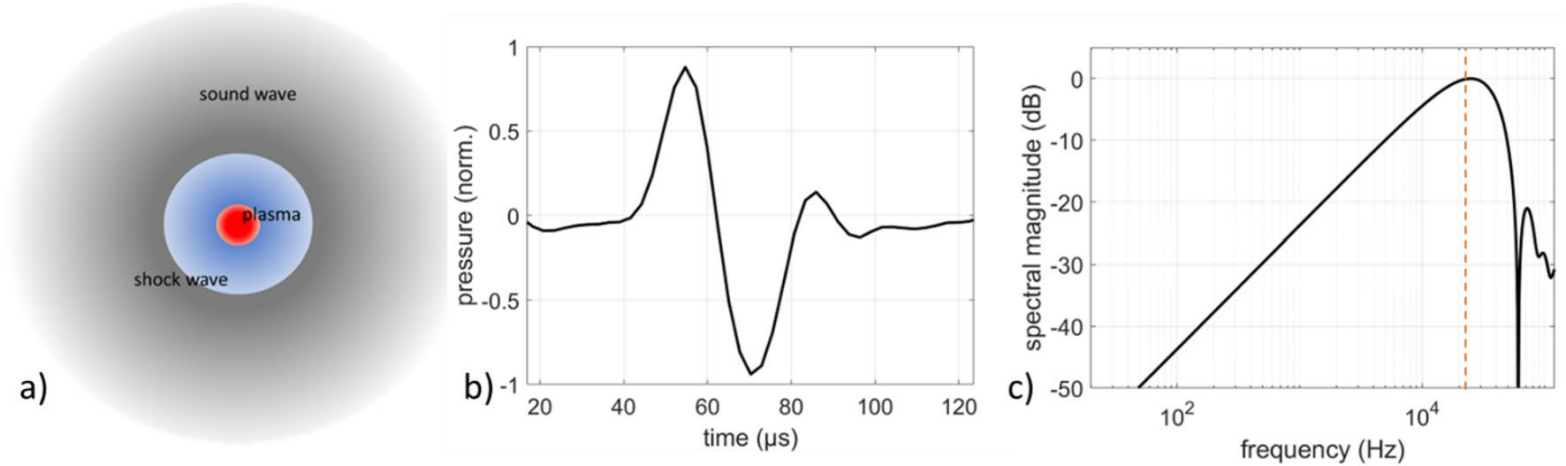
(**a**) Generation of a pressure wave following laser breakdown or laser ablation, initially propagating at supersonic velocity as a shock wave and progressively transforming into a linear sound wave, (**b**) typical pressure profile of an acoustic N-pulse generated by ablation of stainless steel via 532 nm, 10 ns, 1 mJ laser pulses and (**c**) frequency profile of the acoustic N-pulse obtained via DFT. Orange dashed line marks the bandwidth limit of the acoustic measurement system (20 kHz).

Laser-sound generation of complex continuous signals is based on optoacoustic transduction following laser-induced plasma generation by short or ultrashort high intensity laser pulses. For gas targets, e.g., ambient air, the non-linear interaction of such pulses with the neutral gas initially generates a hot electron cloud through photon absorption and electron–electron interactions. The free electrons interact with the colder ions and molecules in the excited volume and transfers energy to them, resulting in rapid and localized thermalization of the gas. The consequent thermal expansion and elastic collapse of the thermalized gas volume lead to the emission of a shock wave that exhibits a characteristic N-pulse shape, with the positive part corresponding to the expansion phase and the negative to the collapse phase. As the shock wave propagates away from the source, it gradually becomes a linear acoustic wave, as shown in Fig. 1a.

For solid targets, the generated acoustic wave has the same characteristic shape in the time-domain, however the physical mechanism leading to the initial shock formation is different. Plasma generation and the resulting heating causes the material near the surface to sublimate in a process known as laser ablation^19^. The escaping particles collide with the surrounding air particles, which are forced away from the surface of the material. This rapid disposition and consequent relaxation of the air molecules leads to the generation of pressure and the emission of a shock wave with the characteristic N-pulse shape. A typical acoustic N-pulse generated by ablation of stainless steel via 532 nm, 10 ns, 1 mJ laser pulses is shown in Fig. 1b). Laser ablation can be achieved with a fluence of the order of 1 of 10^8^–10^10^ W/cm^2^, which is well below the value required for breakdown in ambient air (~ 10^11^–10^12^). As will be shown in the next Section, the reduction of the breakdown threshold is exploited here to lower the laser intensity required for the proof-of-principle experiments on laser-sound reproduction presented here.

In the frequency domain, the laser-plasma acoustic pulses exhibit a 1st order high-pass profile up to a spectral peak, after which the spectral content drops with frequency. The frequency spectrum of the acoustic pulse of Fig. 1b) obtained via discrete Fourier transform (DFT) is shown in Fig. 1c), where the characteristic high pass profile is apparent up to 20 kHz, which is the bandwidth limit of the acoustic measurement system (see “Methods” section). Importantly for sound generation in the audible range, acoustic pulses generated by tightly focused nanosecond laser pulses with energies of several tens of millijoules exhibit spectral peaks in the low ultrasounds (20–40 kHz), while a significant fraction of the acoustic energy lies within the audible range. A thorough analysis of the acoustic spectra of LPSSs generated under such conditions can be found in^5,6^.

### Pulse amplitude modulation

In traditional pulse amplitude modulation (PAM), the information of the modulating signal is encoded as a stream of pulses with constant width and an amplitude proportional to the instantaneous amplitude of the input signal. Assume an analog input signal $x\left(t\right)$, where $t$ is the time variable, and a time interval ${T}_{0}$ so that $x(n{T}_{0})$ is the amplitude of $x(t)$ at the time instances $n{T}_{0},$ with *n* = 0, 1, 2 …. The PAM signal ${y}_{\text{PAM}}\left(t\right)$ constitutes a pulse train ${s}_{p}\left(t\right)$ with a pulse-to-pulse distance ${T}_{0}$ and amplitude of the nth pulse equal to $x(n{T}_{0})$. To express the PAM signal mathematically, we multiply the input signal by a periodic train of impulses with period ${T}_{0}$:

1 $${x}_{s}\left(t\right)=x\left(t\right)\sum_{m=-\infty }^{\infty }\delta \left(t-m{T}_{0}\right)$$

where $\delta \left(t\right)$ is the Cronecker Delta function, so that:

2 $${x}_{s}\left(t\right)=\left\{\begin{array}{ll}x\left(m{T}_{0}\right), &\quad t=m{T}_{0}\\ 0, &\quad otherwise\end{array}\right.$$

Then, the signal ${x}_{s}\left(t\right)$ is convolved with the time profile of a single pulse $p\left(t\right)$, resulting to the PAM signal:

3 $${y}_{\text{PAM}}\left(t\right)={x}_{s}\left(t\right)*p\left(t\right)=\left[x\left(t\right)\sum_{m=-\infty }^{\infty }\delta \left(t-m{T}_{0}\right)\right]*p\left(t\right)$$

The frequency spectrum of ${y}_{\text{PAM}}\left(t\right)$ is calculated by the Fourier transform:

4 $${Y}_{\text{PAM}}\left(\omega \right)={X}_{s}\left(\omega \right)P(\omega )$$

where $\omega$ is the angular frequency. The Fourier transform ${X}_{s}\left(\omega \right)$ of ${x}_{s}(t)$ is given by:

5 $${X}_{s}\left(\omega \right)=\frac{1}{{T}_{0}}\sum_{k=-\infty }^{\infty }X(\omega -k{\omega }_{0})$$

Finally:

6 $${Y}_{\text{PAM}}\left(\omega \right)=\frac{1}{{T}_{0}}\sum_{k=-\infty }^{\infty }X(\omega -k{\omega }_{0})P(\omega )$$

The spectrum ${Y}_{\text{PAM}}\left(\omega \right)$ is the sum of infinite copies of the input spectrum $X(\omega )$ shifted by $k{\omega }_{0}$ and weighted by $P\left(\omega \right)$. If the input signal is bandlimited in $f\in \left[0,{f}_{0}\right)$ so that the copies $X(\omega -k{\omega }_{0})$ do not overlap, the spectrum of the PAM signal in the baseband is given by Eq. (6) for $k=0$:

7 $${Y}_{\text{PAM}}\left(\omega \right)=\frac{1}{{T}_{0}}X(\omega )P(\omega )$$

This is the spectrum of the input signal weighted by the spectral profile of the N-pulse. Unlike most PAM implementations that use rectangular pulses, the laser-sound system reproduces the typical N-pulses ${p}_{N}(t)$ with the 1st order high-pass spectral profile ${P}_{N}\left(\omega \right)$ in the band of interest, as is well-known from existing works^6,13^. Hence, the spectrum of the PAM-based laser sound system in the baseband is a high pass filtered version of the input spectrum:

8 $${Y}_{\text{LS}-\text{PAM}}\left(\omega \right)\sim X\left(\omega \right){P}_{N}(\omega )$$

## Experimental platform

**Figure 2:**
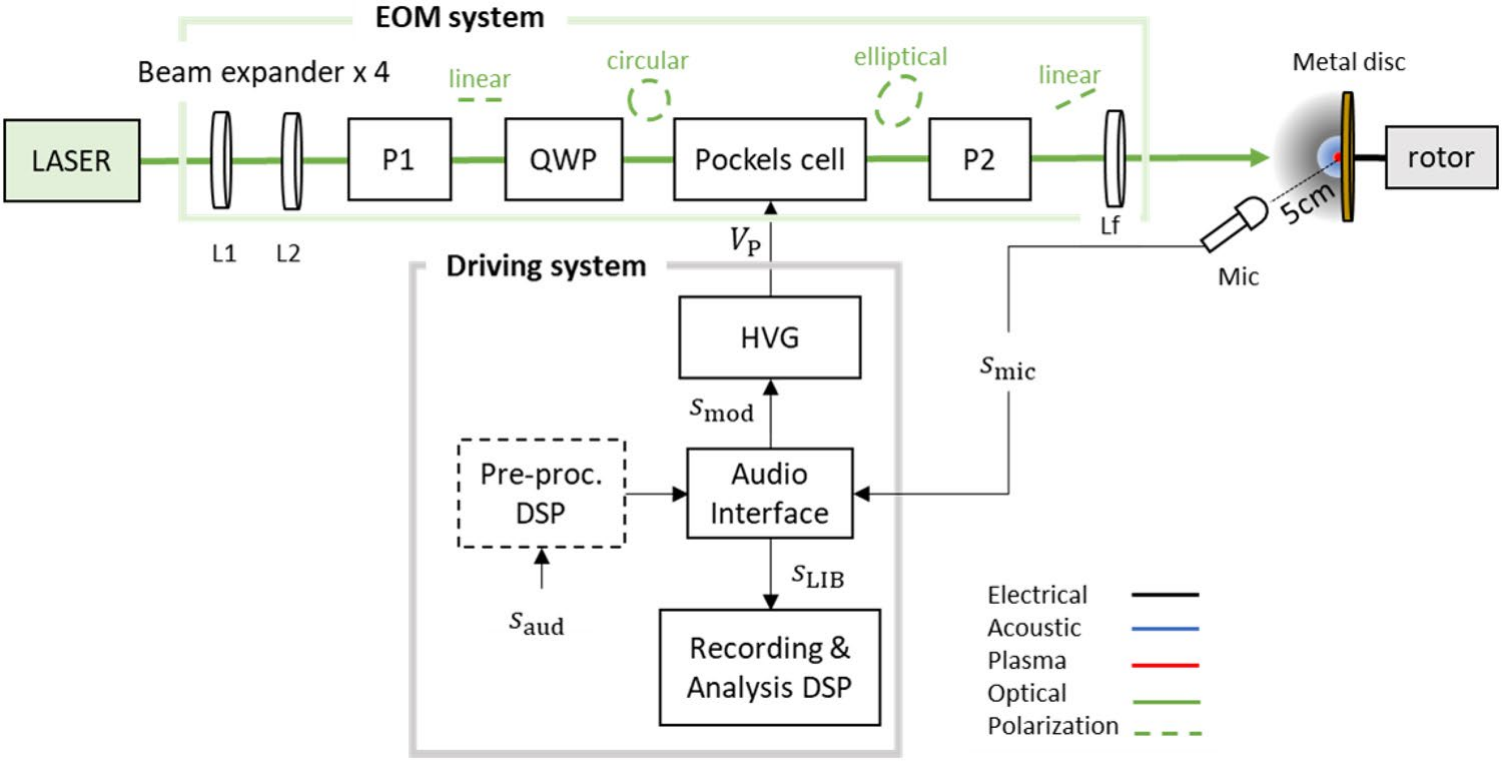
Block diagram of the LS-PAM prototype platform.

The basic structure of the PAM laser-sound system is shown in Fig. 2. Laser pulse amplitude modulation is achieved by an electro-optic modulator (EOM) based on the electro-optic Kerr effect. Initially, the laser beam is expanded by 4 times (L1, L2) in order to achieve stronger focusing on the target and is then directed into a polarizer (P1) that removes any unpolarized components. Then a quarter wave plate (QWP) converts the linear polarization into circular. Consequently, the beam enters the Pockels cell, a crystal whose birefringence is electrically controlled via a high voltage generator. During propagation inside the Pockels cell, the pulse polarization is shifted to a desired polarization state depending on the amplitude of the applied voltage. By exiting the Pockels cell, the pulse is directed into a second polarizer (P2) that blocks the horizontally polarized components. As a result, the amount of light that reaches the focusing lens (Lf) depends on the amount of vertically polarized light at the exit of the Pockels cell.

As shown in Fig. 2, the high voltage generator (HVG) that controls the Pockels cell is driven by a function $f\left(x\left(t\right)\right)$ of the analog audio signal ${s}_{\text{aud}}$, delivered through an audio interface. This results in the laser pulse train and, consequently, the reproduced acoustic pulse train, being correlated with the input audio signal. Particularly, the possible amplitude levels of the laser pulses are theoretically infinite and continuous, which in turn results to infinite and continuous amplitude levels of the generated acoustic pulses, leading to an analog reproduced audio signal. Moreover, by choosing a linear function ${f}_{\text{lin}}\left(x\left(t\right)\right)$, the modulation of the optical pulse energy is directly proportional to the instantaneous amplitude of $x(t)$, hence producing a linear analog PAM optical pulse stream. However, as is also shown in the “Results” section, this is not an optimal choice because the complex and highly non-linear physical processes resulting to laser-plasma sound generation (see also^6^) introduce non-linearities in the LS-PAM system’s acoustic response. This becomes evident by considering the LS-PAM system as a three-step conversion process:

$${\text{Electrical signal}}\;\left( {\text{audio input}} \right) \to {\text{optical signal}}\;\left( {\text{laser pulse stream}} \right) \to {\text{acoustic signal}}\;\left( {\text{acoustic pulse stream}} \right).$$

In the first step, the audio input $x\left(t\right)$ is used to modulate the amplitude of the laser pulses, so that for the nth laser pulse ${p}_{\text{laser}}\left(n\right)$, it holds:

$${p}_{\text{laser}}\left(n\right)\sim f\left(x\left(n{T}_{0}\right)\right)$$

The electro-optic modulation system allows for precise control of the laser pulse amplitude so that the relation between the instantaneous amplitude $x\left(n{T}_{0}\right)$ of the audio signal at time $t= n{T}_{0}$ and the amplitude of the *n*th laser pulse ${p}_{\text{laser}}\left(n\right)$ can be considered linear and time independent. Also, it is well-known that the Pockels cell has a response in the picosecond scale, rendering any changes in its polarization state instantaneous compared to the time scales of the modulation. However, for the next step of the process, the conversion of the laser pulse to an acoustic pulse:

$${p}_{\text{laser}}\to {p}_{N}$$

is strongly non-linear. Hence, the selection of a linear function ${f}_{\text{lin}}\left(x\left(t\right)\right)$ is not optimal and leads to acoustic non-linearities in the reproduced signal. The selection of the optimal function requires an evaluation of the ${p}_{\text{laser}}\left(n\right)\to {p}_{N}(n)$ relation, which is here carried out experimentally. Inversion of the ${p}_{\text{laser}}\left(n\right)\to {p}_{N}\left(n\right)$ relation leads to improved acoustic reproduction with suppressed harmonic distortion. Further details on the Pockels cell driving are presented in the “Results” section.

## Results

In the “Results” section, experimental results on the ability of the analog PAM laser-sound system to reproduce single sinewaves, two tone signals and sine sweep signals are presented. The impulse and frequency response of the system are obtained and compared to the simulated response. The possibility for system response equalization by input signal pre-filtering is also experimentally demonstrated for the two-tone signals. Two different control mechanisms, a linear and a non-linear, are investigated and compared in terms of generated harmonic distortion.

### Linear control

**Figure 3:**
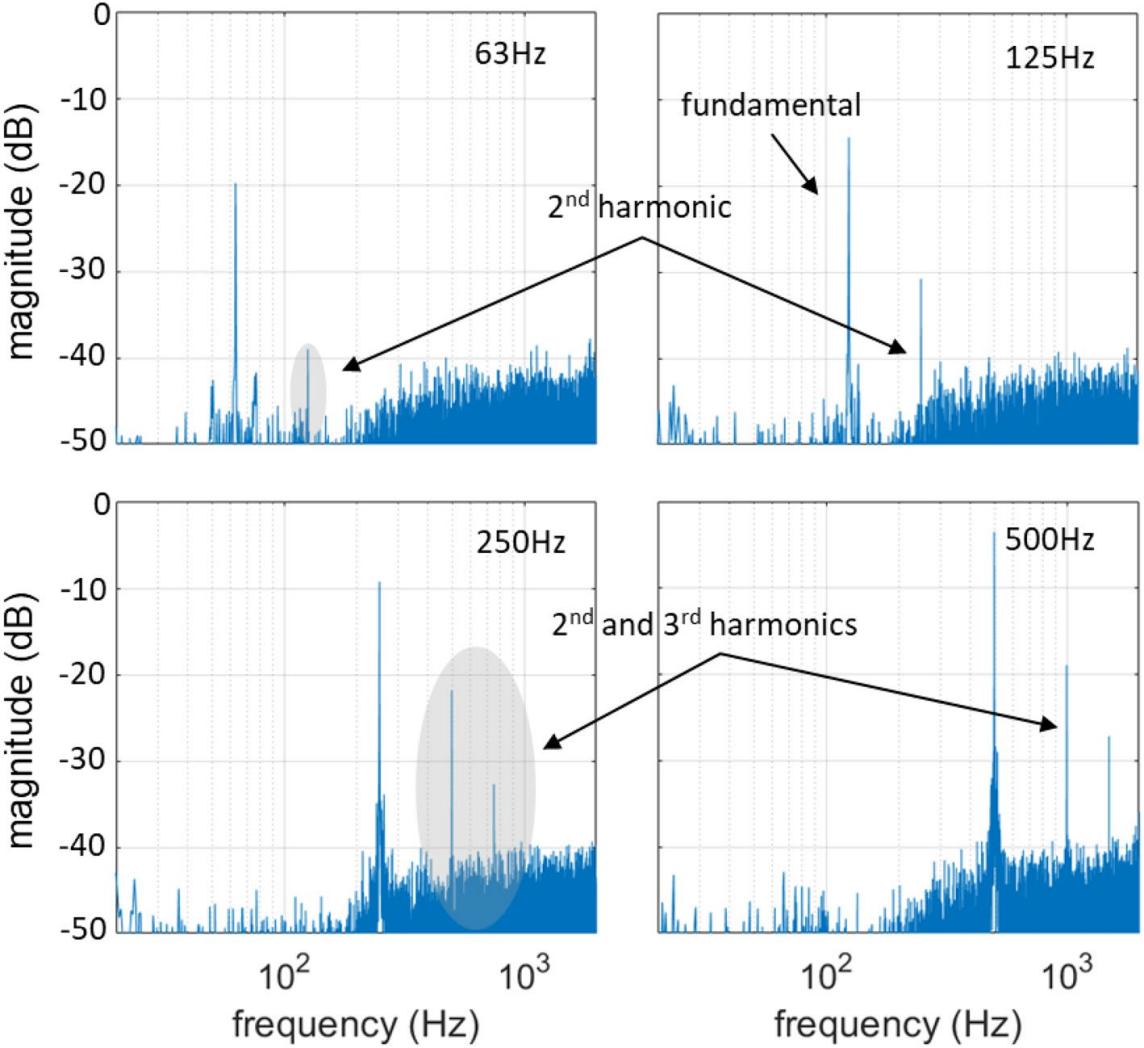
Measured sinewave signals with frequencies 63, 125, 250 and 500 Hz reproduced by the linearly driven PAM laser-sound system.

Figure 3 shows the experimentally measured spectra of discrete sinewaves with frequencies 63, 125, 250 and 500 Hz, respectively, for a linear driving function ${f}_{\text{lin}}\left(x\left(t\right)\right)$. It can be seen that the magnitude of the fundamental frequencies of the sinewaves increases by 6 dB when the frequency is doubled. This is in accordance with the linear behavior of the system, characterized by the 1st-order high pass profile, As described in the model presented in the subsection “pulse amplitude modulation”. The higher harmonics also increase with increasing fundamental frequency, while the background noise remains the same, as expected. The measured spectra include additive noise, mainly originating from the rotor of the metal target. The contribution of the rotor to the background noise was measured to be approximately 55 dB-SPL. Another source of noise in the measured signal is a random deviation in the amplitude of the generated acoustic pulses from the expected. Despite the high repeatability of laser-plasma sound generation, pulse-to-pulse repeatability in the presented experiments was compromised by the low available optical power, which was close to the breakdown threshold. This, in combination with the use of the metal disc, whose progressive surficial degradation due to ablation lead to uncontrollable irregularities in the ablation conditions, resulted to a generally increased noise in the measurements. Higher optical power and careful design of the ablating target, or optimally the transduction in the air, would significantly improve the acoustic performance of the system.

**Figure 4:**
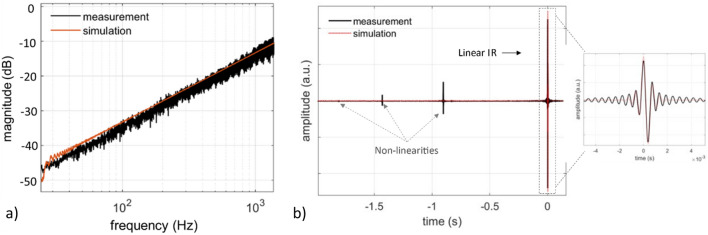
Measured and simulated (**a**) frequency response and (**b**) impulse response of the linearly driven PAM laser-sound system.

Moreover, higher order harmonics are present in the measured signals, especially the 2nd harmonic, while the 3rd harmonic is also apparent above the noise floor for the 250 and 500 Hz sinewaves. The harmonic distortion originates in the non-linear relation between the amplitude of the optical pulses and the resulting amplitude of the acoustic pulses. A preliminary investigation of the non-linearities of the PAM laser-sound system is presented in the next subsection. Figure 4a shows the experimentally measured frequency response of the system in comparison to the simulated frequency response, whereby a very good agreement can be observed. Both curves have the characteristic 1st-order high pass profile, as predicted by the computational model of Eq. (8), for a flat input spectrum $X\left(k\right)=1$:

9 $${H}_{\text{LS}-\text{PAM} }(\omega )={P}_{N}(\omega )$$

The main difference between the two curves is the apparent additive noise in the measured signal.

The experimentally evaluated and simulated Impulse Responses (IRs) of the system are presented in Fig. 4b, where amplitude normalization for the two curves is carried out so that the energies of the two signals are equal. In the IR signal, three distinct features can be observed, with the prominent feature corresponding to the linear response and the preceding features corresponding to the non-linearities of the system, as described in^20^. From Eq. (9) and for $x\left(t\right)=\delta (t)$ we obtain the linear impulse response of the system:

10 $${h}_{\text{LS}-\text{PAM}}(t)={p}_{N}(t)$$

which effectively is the N-pulse pressure profile. In the zoomed window of the measured IR, a central N-pulse can be observed along with lateral ripples. The ripples originate from the frequency range of the input sine-sweep, which is limited to 2 kHz compared to the 192 kHz bandwidth available at the used 384 kHz sampling rate.

### System response equalization

**Figure 5:**
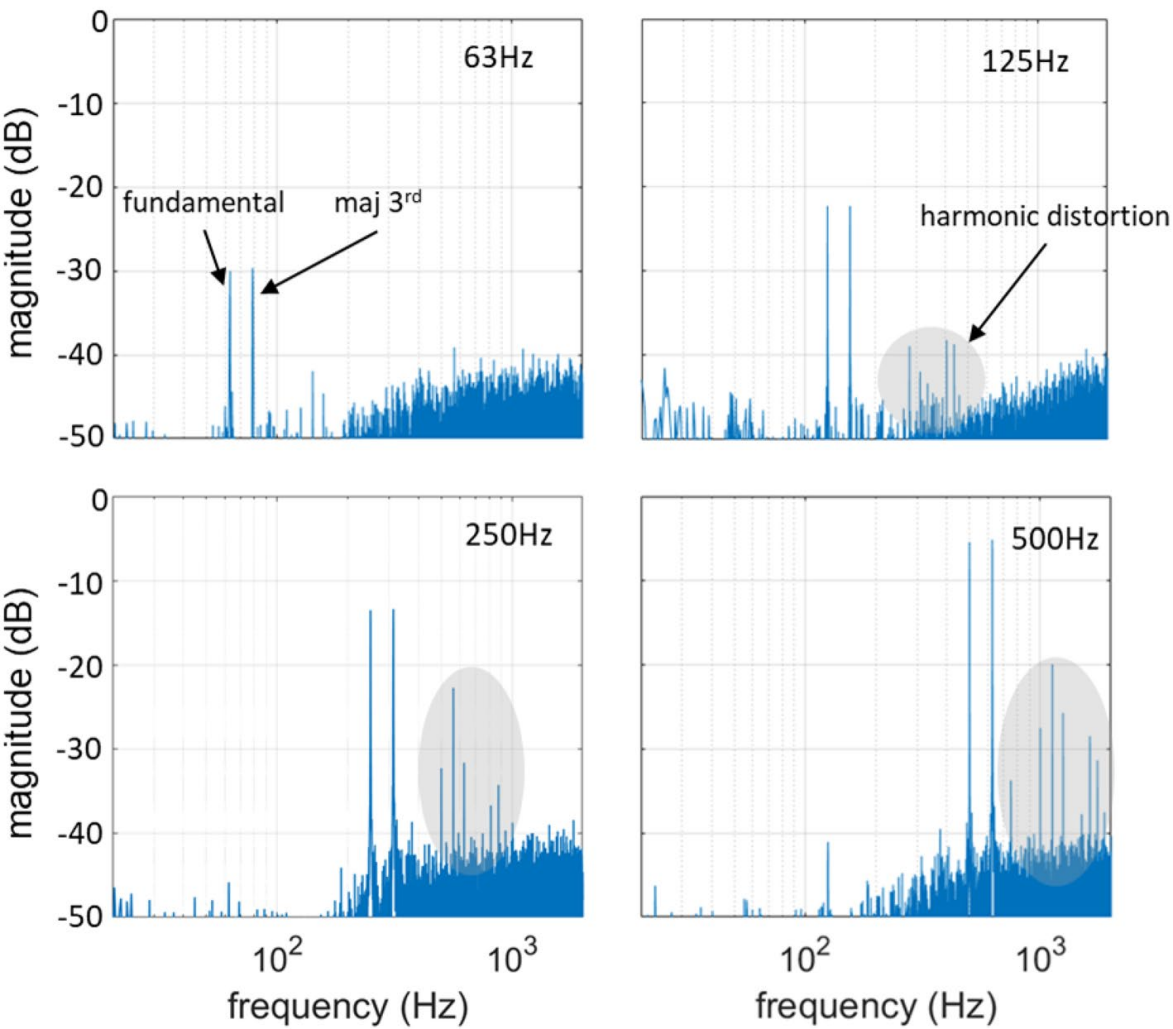
Measured two-tone signals with fundamental frequencies 63, 125, 250 and 500 Hz generated by the linearly driven PAM laser-sound system.

As it was computationally demonstrated for the ΣΔ-based laser-sound system^5,6^, the high pass frequency response of the LS-PAM system can be equalized by pre-filtering of the input signal with a 1st-order low pass filter (see also “Signal processing” subsection in “Methods” section). Here, an experimental demonstration of system equalization is presented. In Fig. 5a–d the measured response of the LS-PAM system is presented for reproduction of two-tone signals with a distance of the musical interval of a major 3rd and fundamental frequencies 63, 125 250 and 500 Hz, respectively. It can be seen that the spectral magnitudes at ${f}_{0}$ and ${f}_{3\text{maj}}$ are practically equal (less than 0.3 dB difference), while without pre-filtering the spectral magnitude at ${f}_{3\text{maj}}$ would be $20{\text{log}}_{10}\frac{5}{4}\cong 2 \,\text{dB}$ higher than at ${f}_{0}$. It is noted that, after equalization, each two-tone signal was individually normalized to the maximum possible amplitude, so that the best signal to noise ratio (SNR) to be separately achieved for each signal.

### Non-linear control

**Figure 6:**
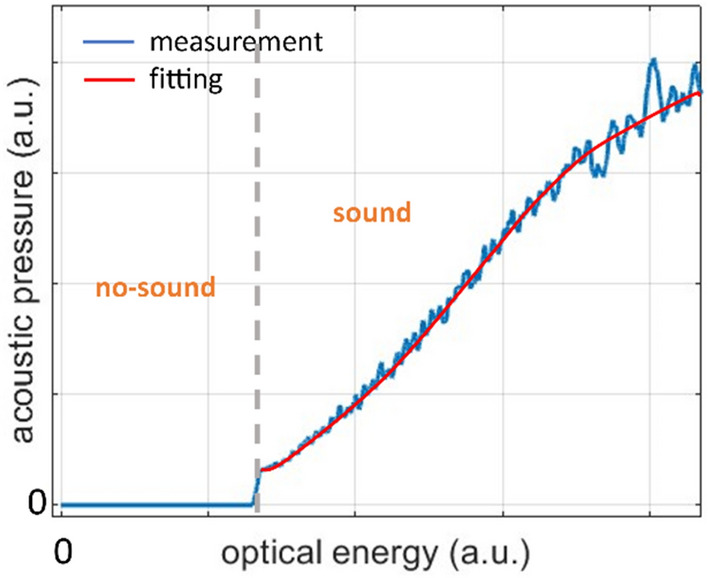
Measured curve of the relation between laser pulse energy and acoustic pressure.

The acoustic performance of the system can be significantly improved by considering the non-linear relation $g(\cdot )$ between optical excitation energy and reproduced acoustic amplitude. This can be done by measuring the reproduced acoustic pulses for a ramp control signal. Note that the relation between the input signal and the laser pulse amplitude is linear so that the two can be used interchangeably for the purposes of the following analysis. The results are shown in Fig. 6, where two main regimes can be identified in the *p*_laser_–*p*_*N*_ curve:

- a sub-threshold—or “no sound”—region, where the optical intensity of the laser pulses is not sufficient to ablate the metal target and hence, no sound is produced,
- a “sound” region with a sigmoid-like profile.

It should be noted that this curve corresponds to the specific laser system and the metal target and hence cannot be considered as general.

**Figure 7:**
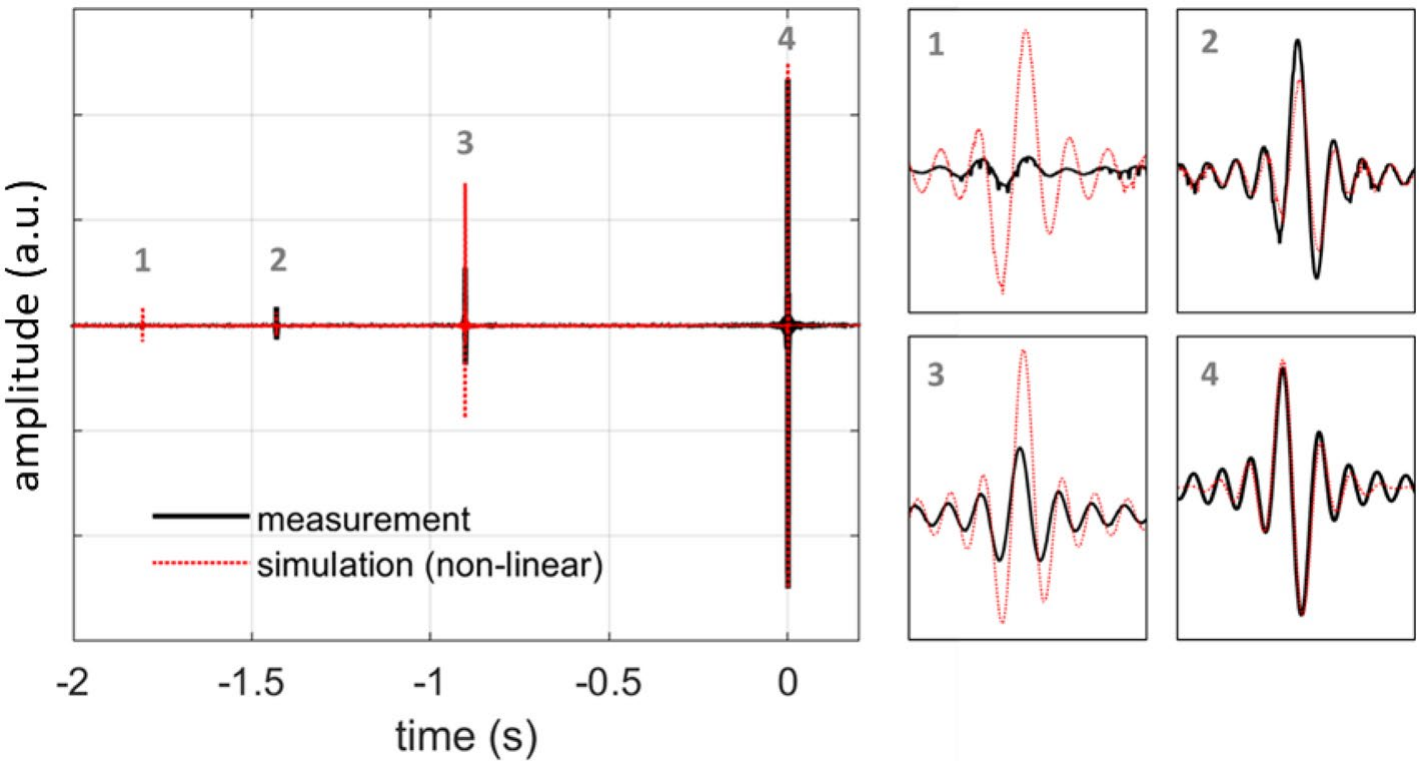
Simulated impulse response of the linearly driven PAM laser-sound system accounting for the non-linear relation between laser pulse energy and generated acoustic pressure. Comparison with the measured impulse response. Insets show details of the linear and non-linear IR features.

The measured non-linear relation can be used to calculate the system’s impulse response via the computational model, by applying $g(\cdot )$ in the calculated PAM signal ${y}_{\text{PAM}}^{sw}\left(t\right)$ resulting from a sine sweep input as:

11 $${y}_{\text{LS}-\text{PAM}}^{sw}\left(t\right)=g\left({y}_{\text{PAM}}^{sw}\left(t\right)\right)$$

where ${y}_{\text{LS}-\text{PAM}}^{sw}\left(t\right)$ is the calculated output of the system when the non-linearity of the *p*_laser_–*p*_*N*_ curve is considered. The result is plotted against the measured IR in Fig. 7, where the curves are again normalized to have the same energy. It can be seen that the model correctly identifies the appearance of the three higher-order harmonics, hence demonstrating the influence of the *p*_laser_–*p*_*N*_ curve on the non-linear behavior of the system. The overestimation of the amplitude of the second harmonic highlights the need for more precise evaluation of the *p*_laser_–*p*_*N*_.

It can be argued that suppression of the higher-order harmonics requires the elimination of the “no-sound” region and the linearization of the “sound” region. For the former, various methods related to the physics of laser-matter interaction could be considered, as for example the use of femtosecond laser pulses or the development of special targets with lower breakdown or ablation thresholds. For the non-linearity of the “sound” region, a general roadmap entails application of the inverse function ${g}^{-1}\left(\cdot \right)$ of the sigmoid curve to the input signal, so that:

12 $${x}^{\prime}\left(t\right)={g}^{-1}\left(x\left(t\right)\right)$$

It can be easily seen that such a predistortion of the input signal theoretically leads to a distortion-free PAM signal reproduced by the laser-sound system:

13 $${y}_{\text{LS}-\text{PAM}}\left(t\right)\sim g\left({{x}^{\prime}}_{s}\left(t\right)\right)*{p}_{N}\left(t\right)=g\left({g}^{-1}\left(x\left(t\right)\right)\right)*{p}_{N}\left(t\right)=x(t)*{p}_{N}\left(t\right)$$

Here, preliminary experimental results by measurements using predistortion of the input signal based on the measured curve of Fig. 6 are presented. It should be noted that, since the curve of Fig. 6 is valid only within the particular amplitude range of the input signal, effective suppression of the system non-linearities requires that the pre-distorted signal has the same amplitude range.

**Figure 8:**
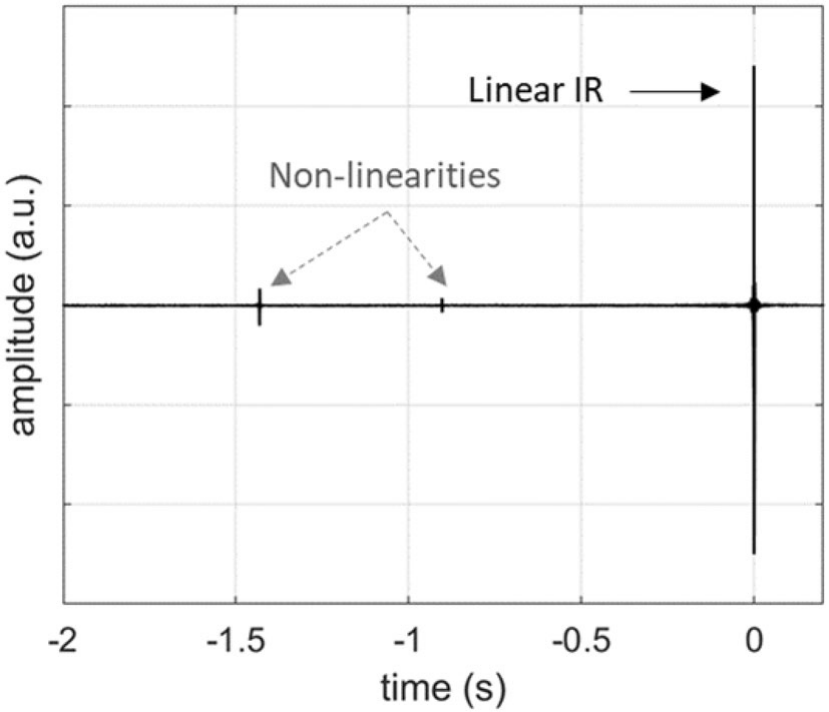
Impulse response of the non-linearly driven PAM laser-sound system.

**Figure 9:**
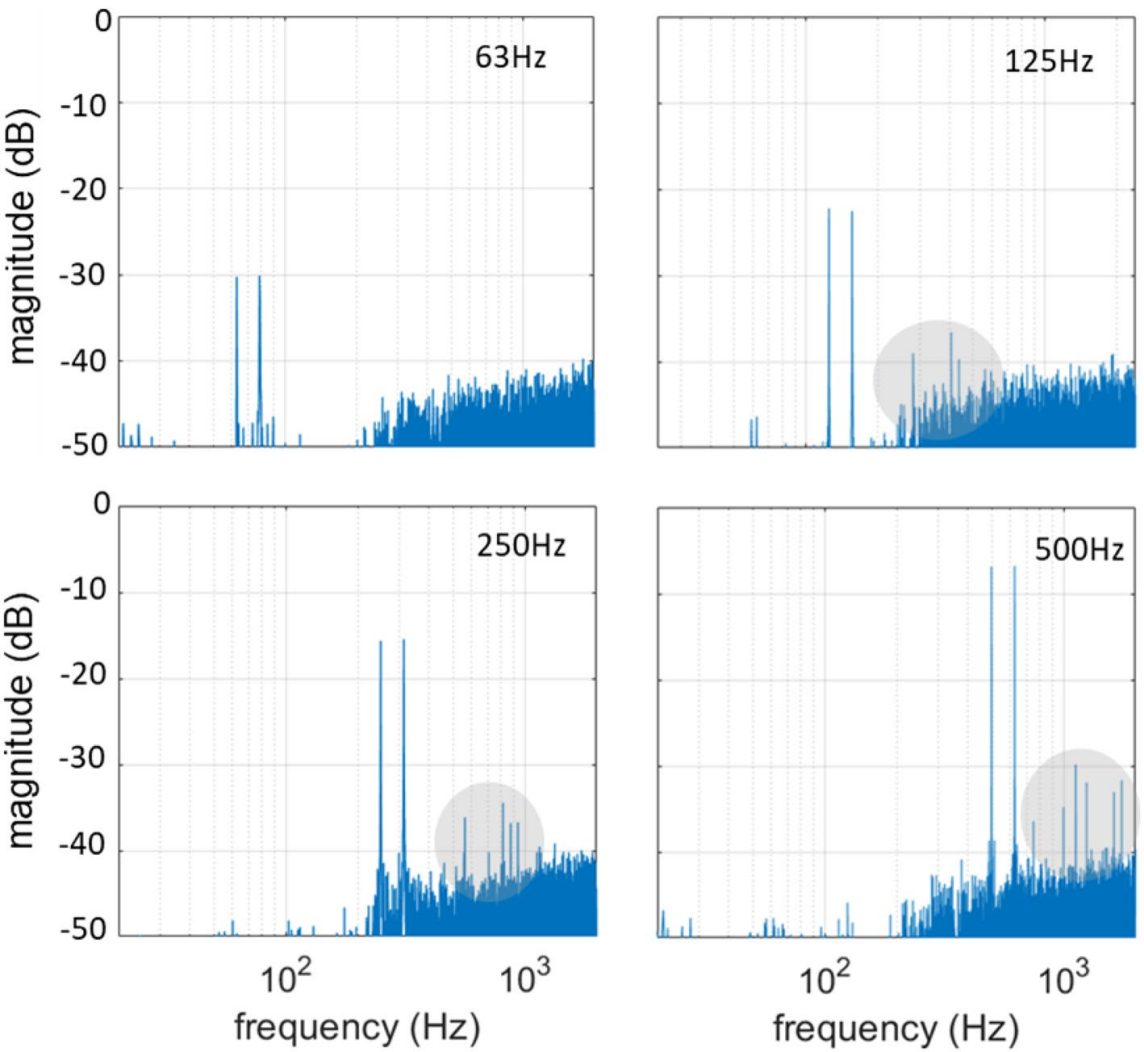
Measured two-tone signals with fundamental frequencies 63, 125, 250 and 500 Hz reproduced by the non-linearly driven PAM laser-sound system.

Figures 8 and 9 show the measured impulse response and two-tone signals reproduced by the system, respectively. From the impulse response it becomes immediately apparent that the second harmonic, which is the strongest non-linear feature when linear control is used, is almost eliminated, while the third harmonic is mostly left unaffected. Quantification of the harmonic energy in the two curves shows a reduction of about 85% for the non-linear control method. This fact is also reflected in the spectra of the two-tone signals of Fig. 9. Particularly, for the 63 Hz fundamental frequency, no harmonic distortion is observed above the noise level, while for the 125, 250 and 500 Hz, the harmonic distortion is significantly suppressed compared to that of Fig. 5b–d. Particularly for the fundamental frequencies of 250 and 500 Hz, the magnitude of the higher harmonics is suppressed by more than 10 dB.

## Discussion

In this work, a laser-sound system based on pulse amplitude modulation was developed and experimentally evaluated for the reproduction of test audio signals. The system effectively constitutes an extension of the original ΣΔ-based laser-sound system towards amplitude modulation without quantization of the permitted acoustic levels. It uses laser-plasma sound generation to form acoustic pulse trains by fast nanosecond laser excitation on a metallic target. Modulation of the laser radiation was achieved using an electro-optic modulator based on a Pockels cell. It was shown that the PAM laser-sound system achieves reproduction of acoustic signals within an intended band of interest by using a laser repetition rate two times the Nyquist frequency of the band. Experiments were carried out for reproduction of single sinewaves, two-tone signals and sine-sweep signals at a repetition rate of 8 kHz. This was only restricted by the specifications of the available laser unit but can be raised to 40 kHz or more in order to cover the complete audible spectrum.

For the control of the laser pulse amplitude modulation from the audio input signal, two different driving approaches, a linear and a non-linear, were investigated. For the linear control, noticeable harmonic distortion was observed in the reproduced acoustic signals, which was associated with the non-linear relation between laser pulse and acoustic pulse energy. Owing to its pulsed nature, laser-based sound reproduction allows for simple experimental evaluation of this non-linearity, in contrast to non-linearities encountered in traditional electromechanical transducers^21^. Here, the non-linear relation was experimentally evaluated by measurements of the reproduced pulse trains for a ramp input signal. The results were used in computational simulations, which confirmed the experimental findings. Preliminary experimental results from the application of non-linear control aiming to reverse the non-linearity of the system by appropriate predistortion of the input signal, showed significant reduction of the harmonic distortion in the reproduced signal. It is noted that predistortion is a well-established technique used in various applications e.g., in RF amplifiers^22,23^.

In comparison to the previously presented digital ΣΔ-based lased-sound system, the PAM laser-sound system can significantly reduce the required repetition rate of the driving laser to the Nyquist frequency of the input audio signal. This is an important finding, as the repetition rate is a critical factor for the feasibility, efficiency and cost of a fully functional laser-sound system operating in the complete audible range. However, the susceptibility of the PAM laser-sound system to harmonic distortion and noise could impose limitations on the fidelity of the reproduced audio signals. This could potentially restrict its applicability to cases where there are no stringent requirements for reproduction accuracy. A full comparison of the performance of the ΣΔ and PAM laser-sound systems for high-fidelity audio reproduction will be carried out in the future.

## Methods

### Experiments

**Figure 10:**
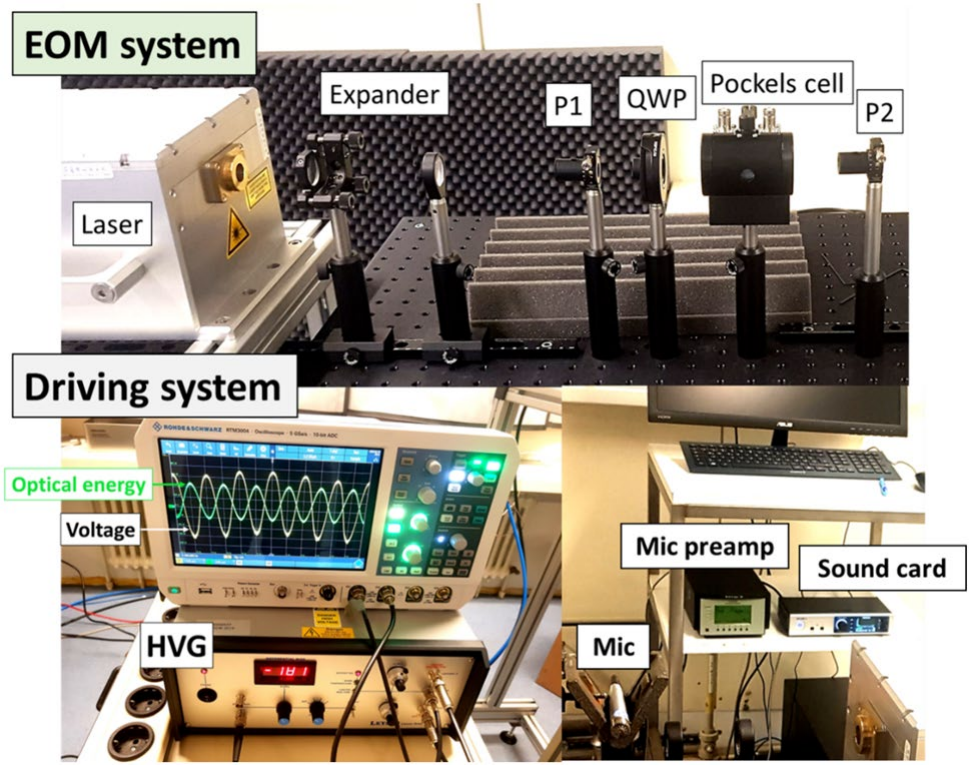
Picture of the prototype LS-PAM experimental platform, where the EOM and driving systems can be seen.

A picture of the prototype LS-PAM experimental platform can be seen in Fig. 10. It is based on an Nd:YAG laser (IS-200-2-L, EdgeWave, Germany) capable of emitting pulses with 532 nm wavelength, 10 ns duration and 1 mJ energy at a repetition rate of 8 kHz. In the experiments, a fixed repetition rate of 8 kHz was utilized. The acoustic streams were captured recorded by a special microphone (4192, B&K, Denmark, Germany) with a high dynamic range of 19–162 dB and frequency response spanning from the low infrasonic frequencies to the high audible frequencies $f\in [3\,{\text{Hz}},\; 20\,{\text{kHz}}]$ using a microphone preamplifier (2690-0S2, B&K, Denmark). The microphone was placed at a distance of 5 cm from the target point. The microphone signal was sampled by an audio interface (Adi-2 Pro, RME, Germany) at ${f}_{s}=384\, {\text{kHz}}$ with a 24-bit resolution. Recording was done via the Audacity software^24^ enhanced by the Aurora plugin^25^.

Optoacoustic transduction was done here by focusing the laser pulses on a metal disc, which allowed for a significant reduction of the required optical energy compared to focusing into ambient air. This was necessary due to limitations in the available optical power of the laser. The disc was mounted on a rotor rotating at approximately 300–500 rpm (7450 ES, EBM-Papst, Germany), in order to avoid repeated consecutive ablation on the same spot, which would lead to rapid localized degradation of the material with detrimental effects on the reproduced sound. It should be noted that the main acoustic characteristics of the system are not affected by the use of the rotating metal target, as the pressure profile of the acoustic pulses generated by laser ablation on metals is identical to that of laser-induced breakdown. This fact becomes apparent from the pressure profile and frequency spectra of the typical ablation-generated acoustic N-pulse shown in Fig. 1b and c.

### Signal processing

**Figure 11:**
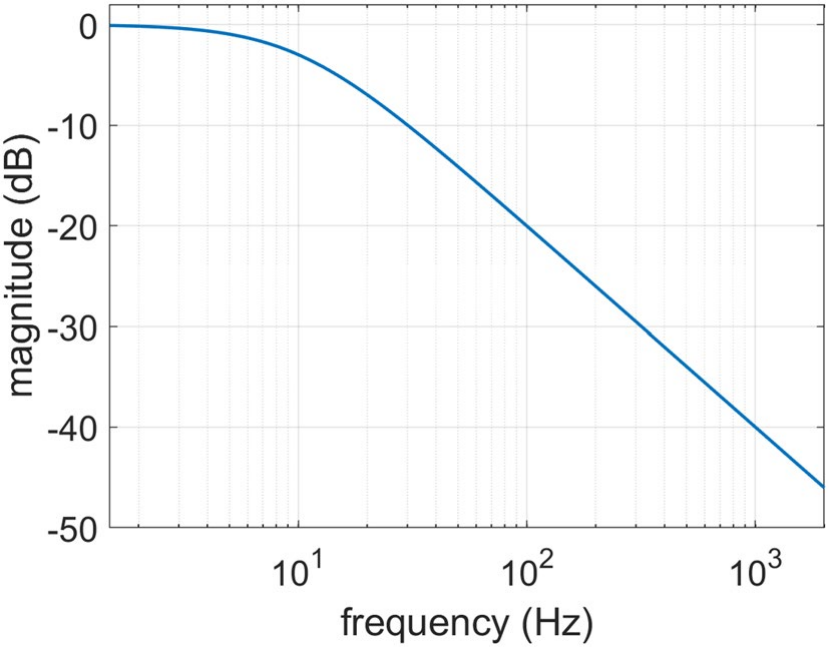
Frequency response of the 1st order low pass filter used for equalization of the LS-PAM system.

The input signals used here for the evaluation of the system are single sine waves with frequencies 63, 125, 250 and 500 Hz (octave-bands central frequencies) as well as two-tone signals containing an octave-band central frequency and a frequency in the harmonic interval of a major 3rd. The higher tone ${f}_{3}$ in the major 3rd interval is related to the fundamental tone ${f}_{0}$ according to ${f}_{3}=\frac{5}{4}{f}_{0}$ and is suitable for aural evaluations as it can be easily identified by its characteristic consonance. System equalization was carried out by pre-filtering of the two-tone input signals with a 1st order low pass filter. The filter’s frequency response is shown in Fig. 11.

Moreover, logarithmic sine sweeps ${x}^{sw}\left(t\right)$ from 20 Hz to 2 kHz are used to evaluate the impulse and frequency response of the system. The impulse response ${h}_{\text{PAM}}\left(t\right)$ is obtained by convolution of the measured signal ${y}_{\text{LS}-\text{PAM}}^{sw}\left(t\right)$ with the inverse filter ${h}_{\text{inv}}\left(t\right)$ of the logarithmic sweep:

14 $${h}_{\mathrm{LS}-\text{PAM}}\left(t\right)={y}_{\mathrm{LS}-\text{PAM}}^{sw}\left(t\right)*{h}_{\text{inv}}\left(t\right)$$

where

15 $${x}^{sw}\left(t\right)*{h}_{\text{inv}}\left(t\right)=\delta \left(t\right)$$

The frequency response of the PAM laser-sound system is derived from the impulse response by DFT:

16 $${H}_{\text{LS}-\text{PAM}}\left(k\right)=\mathcal{F}\left\{{h}_{\text{LS}-\text{PAM}}\left(t\right)\right\}$$

It should be noted that, due to the nature of laser-plasma sound generation, it is impossible to control the sign of the generated N-pulses. Simply, it is impossible to produce “negative” N-pulses as a typical PAM signal would require and hence unipolar PAM needs to be used. For this reason, a unipolar input signal is generated by adding a DC component to the input signal equal to the minimum value of the signal:

17 $${x}_{u}\left(t\right)=x\left(t\right)+\text{min}\left(x\left(t\right)\right)$$

Finally, the measured signals (single, two-tone sinewaves and sine sweep signals) were post-processed to reduce the ambient noise and acoustic distortion due to the room reverberation. The post-processing procedure was held in two stages: (1) windowing of the measured signals in the time domain so that each N-pulse is multiplied by a Tukey window, with the top-flat part of the window corresponding to the duration of the pulse and its decaying part eliminating the distortions occurring in the time intervals between two consecutive pulses, (2) spectral subtraction of a smoothed noise power spectrum derived from a recorded noise profile from the measured signal spectrum on the achievable in-band spectral range of the measurement.

## Ethics declarations

The University of Patras and the Karlsruhe Institute of Technology have been granted a relevant patent (DE102021200293) by the German Patent and Trade Mark Office, in which the authors K.K. B.S. and D.T. are included as inventors.

## Data Availability

The datasets used and/or analyzed during the current study available from the corresponding author K.K. on reasonable request.
